# Effects of Preoperative Nutritional Status and Lymphocyte Count on the Development of Early-term Atrial Fibrillation After Coronary Artery Bypass Grafting: A Retrospective Study

**DOI:** 10.21470/1678-9741-2023-0366

**Published:** 2024-05-13

**Authors:** Seyhan Yilmaz, Sabür Zengin, Ahmet Cumhur Dulger

**Affiliations:** 1 Department of Cardiovascular Surgery, Giresun University Faculty of Medicine, Giresun, Turkey; 2 Department of Gastroenterology, Giresun University Faculty of Medicine, Giresun, Turkey

**Keywords:** Coronary Artery Disease, Nutrition Assessment, Atrial Fibrillation, Lymphopenia, Cardiopulmonary Bypass

## Abstract

**Introduction:**

Although there are publications in the literature stating that parameters related to the nutritional status of patients are associated with the clinical outcomes of those with coronary artery disease, it is also stated that there is insufficient data on the relationship between nutritional indices and long-term outcomes and major adverse cardiovascular events in patients undergoing isolated coronary artery bypass grafting.

**Methods:**

This retrospective study was conducted with patients who underwent isolated elective on-pump coronary artery bypass grafting in our hospital. Patients who underwent emergency coronary artery bypass grafting or those with known atrial fibrillation in the preoperative period were excluded. Patients were analyzed and compared in two groups according to the development of postoperative atrial fibrillation.

**Results:**

The data of 93 coronary artery bypass grafting patients (71 [76%] males) with a mean age of 62.86 ± 9.53 years included in the study were evaluated. Both groups had similar preoperative ejection fraction value, hemoglobin level, age, number of distal bypasses, and postoperative mortality rates. Although the mean cardiopulmonary bypass and aortic cross-clamping times were higher in Group 1, they were not statistically significant. In our study, the mean prognostic nutrition index value was 51.76 ± 3002.

**Conclusion:**

According to our study results, there was no statistically significant difference between prognostic nutrition index values and the development of atrial fibrillation after coronary artery bypass grafting, which is similar to some publications in the literature. We think that it would be beneficial to conduct randomized studies involving more patients on this subject.

## INTRODUCTION

**Table:** 

Abbreviations, Acronyms & Symbols	
AF	= Atrial fibrillation
CABG	= Coronary artery bypass grafting
CAD	= Coronary artery disease
CPB	= Cardiopulmonary bypass
CRP	= C-reactive protein
DM	= Diabetes mellitus
ECG	= Electrocardiogram
EF	= Ejection fraction
IABP	= Intra-aortic balloon pump
PNI	= Prognostic Nutrition Index

Coronary artery bypass grafting (CABG) is still been reported as the recommended treatment for coronary artery disease (CAD)^[1]^, which is a major health problem worldwide, especially in patients with diabetes mellitus or left main coronary disease^[2,3]^. It has been stated that an important complication after CABG is rhythm problems, the most common of which is atrial fibrillation (AF), and its incidence is reported to be 10-40%^[4-6]^. This adverse condition is an important cause of mortality and morbidity after CABG, and it has been reported that advanced age, low ejection fraction (EF), thyroid disease, and chronic obstructive pulmonary disease are among the main known risk factors^[7]^. It has been stated that many factors, such as surgical trauma, use of cardiopulmonary bypass (CPB), non-use of beta-blocker agents, hypoxia, and electrolyte imbalance, have been implicated in the pathogenesis of newly occurring AF after CABG^[5]^. In addition to the fact that the inflammatory process is known to be one of the pathophysiologic factors contributing to the occurrence of AF^[8]^, it has also been reported that a high body mass index increases the development of postoperative AF^[9]^.

Although there are publications in the literature stating that parameters related to the nutritional status of patients are associated with clinical outcomes in those with CAD^[1,10]^ and that they are effective in predicting mortality in patients undergoing CABG, it is also stated that there is insufficient data on the relationship between nutritional indices and long-term outcomes and major adverse cardiovascular events in patients undergoing isolated CABG^[1]^. Although it has been reported that a low lymphocyte count may be a risk factor for poor postoperative outcomes and an indicator of poor survival in the same patient group^[11]^, the Prognostic Nutrition Index (PNI)^[12]^ which is formulated by serum albumin and lymphocyte count and is a marker used for simple and objective assessment of the nutritional status of individuals, may be a risk factor for postoperative AF^[4]^. In addition, even in the presence of similar risk factors, there are significant differences in the development of new AF in some patients, and the reasons for this are still unknown^[13]^.

Our study aimed to assess the factors contributing to the onset of postoperative AF in isolated elective on-pump CABG patients. Additionally, we investigated the impact of preoperative PNI and lymphocyte values on the development of postoperative AF in this patient group. Finally, we discussed our findings concerning the existing literature.

## METHODS

Our study was conducted with patients who underwent isolated elective on-pump CABG in our tertiary care hospital between December 2021 and June 2022. Declaration of Helsinki criteria were followed, and the ethical approval was obtained (Ethics Committee Unit, 2023-215115237). Our retrospective study included patients who underwent elective isolated on-pump CABG in our hospital with available study data. Patients who underwent emergency CABG operation, patients who underwent off-pump CABG operation, patients who underwent cardiac or major vascular surgery simultaneously with CABG operation, patients who underwent another open-heart operation other than CABG operation, patients who previously underwent open-heart surgery, patients with severe left ventricular dysfunction, and patients with known AF or severe cardiac arrhythmia in the preoperative period were excluded. Preoperative and postoperative medical data (preoperative baseline clinical characteristics, comorbidities, laboratory parameters, intraoperative data, and significant postoperative complications) of the patients included in the study were reviewed and recorded from the hospital automation system and hospital file archives. A total of 93 patients included in the study were analyzed and compared in two groups according to the development of postoperative AF - Group 1, patients with postoperative AF; and Group 0, patients without postoperative AF.

Fasting (6-8 hours) venous blood tests and biochemical markers were measured in all patients during hospitalization for the operation. Hematologic parameters and serum albumin, lymphocyte, and creatinine values were measured in the laboratory before CABG. The preoperative PNI value of the patients was calculated using the formula [albumin (g/dl) × 10 + lymphocyte count (permm3) × 0.005] as stated in a publication in the literature^[12]^.

### Coronary Artery Bypass Surgery Procedure

CABG operations were generally made with general anesthesia through standard median sternotomy. CABG was performed under CPB guidance in all patients. In all patients, myocardial protection was achieved with antegrade cold blood cardioplegia from the aortic root with intermittent retrograde cold blood cardioplegia from the coronary sinus, and complete revascularization was made. After the operation, the patients were admitted to the cardiovascular surgery intensive care unit and immediately monitored and followed up for 24 hours with a standard D2 lead. They were extubated when they were sufficiently awake and spontaneously breathing, and there was no abnormality in their blood gas parameters and hemodynamic status. Blood transfusions were administered when necessary (if the hematocrit level was < 24-25%).

New-onset AF after CABG was defined as an episode of AF lasting > 5 minutes, detected (by monitoring or 12-lead electrocardiogram [ECG]) during hospitalization. The patients’ immediate monitoring and daily ECG findings determined the presence or absence of AF.

### Statistical Analysis

We conducted all statistical analyses using IBM Corp. Released 2015, IBM SPSS Statistics for Windows, version 23.0, Armonk, NY: IBM Corp. Continuous variables were expressed as mean ± standard deviation. Where appropriate, categorical variables were compared using the chi-square test and Fisher’s exact test. Continuous variables with normal distribution were compared using independent samples *t*-test. Non-parametric data were compared using the Mann-Whitney U test. A *P*-value ≤ 0.05 was statistically significant.

## RESULTS

The data of 93 CABG patients (71 [76%] males) with a mean age of 62.86 ± 9.53 years included in the study were evaluated in the Cardiovascular Surgery Department of our hospital during the study period. Demographic data of the patients are demonstrated in Table 1. Both groups had similar preoperative EF value, hemoglobin level, age, number of distal bypasses, and postoperative mortality rates. Although the mean CPB and cross-clamping times were higher in Group 1, they were not statistically significant. The characteristics of the patients in Groups 0 and 1 are demonstrated in Table 2. In our study, the mean PNI value was 51.76 ± 30.02, and the data regarding the development of AF according to PNI value and lymphocyte count are shown in Table 3 and Table 4, respectively. All patients in the study group were started on oral beta-blocker therapy in the preoperative period since their hospitalization.

**Table 1:** Demographic data of study patients.

Variable	n=93
Male sex (%)	71 (76%)
Age (years)	62.86 ± 9.53
Weight (kg)	80.01 ± 13.26
DM (%)	54 (%58)
Preoperative EF level	54.87 ± 8.64
Preoperative hemoglobin (g/dL)	13.44 ± 1.74
Preoperative lymphocyte (10^9^ /L)	1.94 ± 0.73
Preoperative albumin (gr/L)	42.15 ± 4.01
Preoperative creatinine (mg/dL)	1.10 ± 1.28
Preoperative CRP (mg/l)	13.69 ± 21.95
Preoperative PNI	51.76 ± 5.48
CPB time (minute)	121.53 ± 28.71
Cross-clamping time (minute)	83.31 ± 22.86
Number of distal bypasses	3.88 ± 0.88
Postoperative AF	25 (27%)
Postoperative IABP requirement (%)	5 (5%)
In-hospital mortality (%)	5 (5%)

AF=atrial fibrillation; CPB=cardiopulmonary bypass; CRP=C-reactive protein; DM=diabetes mellitus; EF=ejection fraction; IABP=intra-aortic balloon pump; PNI=Prognostic Nutrition Index

**Table 2:** Demographic characteristics and laboratory findings of the groups.

Variable	Group 1 (n=25)	Group 0 (n=68)	*P*-value
Male sex (%)	21 (84%)	50 (74%)	0.023
Age (years)	62.84 ± 8.4	62.87 ± 9.97	0.219
Weight (kg)	77.24 ± 10.58	81.03 ± 14.06	0.171
DM (%)	12 (48%)	42 (62%)	0.251
Preoperative EF level	54.80 ± 9.40	54.90 ± 8.41	0.454
Preoperative hemoglobin (g/dL)	13.49 ± 1.83	13.42 ± 1.72	0.539
Preoperative lymphocyte (10^9^/L)	1.84 ± 0.57	1.98 ± 0.78	0.084
Preoperative CRP (mg/l)	8.60 ± 16.84	15.56 ± 23.38	0.478
Preoperative albumin (g/L)	41.72 ± 4.33	42.31 ± 3.91	0.356
Preoperative creatinine (mg/dL)	0.94 ± 0.26	1.15 ± 1.49	0.275
Preoperative PNI	50.91 ± 5.29	52.07 ± 5.55	0.877
CPB time (minute)	131.80 ± 33.50	117.75 ± 26	0.418
Cross-clamping time (minute)	88.96 ± 16.51	81.24 ± 24.56	0.008
Number of distal bypasses	4.04 ± 0.79	3.82 ± 0.91	0.767
Postoperative IABP requirement (%)	2 (8%)	3 (4%)	0.185
In-hospital mortality (%)	2 (8%)	3 (4%)	0.185

CPB=cardiopulmonary bypass; CRP=C-reactive protein; DM=diabetes mellitus; EF=ejection fraction; IABP=intra-aortic balloon pump; PNI=Prognostic Nutrition Index

**Table 3:** Data on postoperative AF development by level of PNI value.

Variable	PNI < 51.76 (n=45)	PNI > 51.76 (n=48)	*P*-value
Postoperative AF (%)	31%	23%	0.081

AF=atrial fibrillation; PNI=Prognostic Nutrition Index

**Table 4:** Data on postoperative AF development by level of lymphocyte count.

Variable	Lymphocyte count < 1.5 (10^9^/L) (n=26)	Lymphocyte count > 1.5 (10^9^/L) (n=67)	*P*-value
Postoperative AF (%)	23%	28%	0.286

AF=atrial fibrillation

## DISCUSSION

It has been reported that CABG is still the recommended treatment method for CAD, especially in diabetic or left main coronary disease patients^[3]^. One of the important complications that develop after CABG is rhythm problems, and the most common one is AF, which is reported to be observed in approximately 50% of this patient group^[4-6]^. The main risk factors for newly AF after cardiac operations, stated as an important cause of mortality and morbidity, are reported to be old age, low EF, and thyroid disease^[7]^. Besides surgical trauma and CPB use, many factors, such as hypoxia and electrolyte imbalances, are responsible for the pathogenesis of newly AF in the postoperative CABG period^[5]^. In addition to the fact that the systemic inflammation is known to be a pathophysiological factor contributing to the occurrence of AF^[8]^, it is also stated that a high body mass index increases the occurrence of postoperative AF^[9]^. In the literature, it is stated that there is a relationship between the nutritional status of patients undergoing isolated CABG and long-term important undesirable events and morbidity, that nutritional degree has a critical role in terms of organ and tissue functions, and that in case of malnutrition, the supply of food required for basal organ functions decreases, and disruption in protein turnover occurs^[1,14]^.

Malnutrition has been reported to be related to bad prognosis, especially in old age patients with chronic diseases such as heart failure or CAD or malignancies^[1,15]^, some markers such as PNI scores have been developed to determine nutritional status, and the relationship between these parameters and cardiovascular diseases has been shown in some studies^[1,10,16]^.

It has been reported that several studies have shown a link between the development of postoperative AF and inflammatory biomarkers^[13,17]^. Surgical stress and CPB were reported as cause of immune response, which is influenced by lymphocytes and other anti-inflammatory factors^[5,12]^. Also, it has been reported that preoperative lymphocyte count is a straight forward inflammatory marker that can be obtained easily, and lymphopenia may indicate poor survival after cardiac surgery and be associated with important undesirable events^[12]^. Preoperative lymphocyte count is reported to be an important predictor of mortality in patients undergoing cardiac surgery, and there is an association between low preoperative lymphocyte count and the length of CPB^[11,18]^. In addition, malnutrition and the severity of cardiovascular disease have been reported to cause preoperative lymphocyte count reduction and dysfunction^[11,19,20]^. In our study, although the mean preoperative lymphocyte count was lower in the group with AF compared to the other group (1.84 ± 0.57 *vs.* 1.98 ± 0.78, respectively, *P*=0.084), it was not statistically significant. In addition, there was no statistical difference between the group with lymphocyte count < 1.5 (10^9^/L) and the group with lymphocyte count > 1.5 (10^9^/L) in terms of the development of new postoperative AF, in our study. In the literature, it is reported that inflammation and immune-based prognostic scores, as well as established pro-inflammatory biomarkers such as C-reactive protein (CRP) levels, predict mortality and morbidity after cardiac surgery^[21,22]^. Although it has been reported that CRP and natriuretic peptides are promising biological markers for the development of AF^[23]^, in the same study, they report that the relationship between preoperative and postoperative CRP levels and postoperative AF development in CABG patients cannot be detected^[21]^. In another study in the literature, CRP levels are high in patients with AF, however, it has been reported that there is no correlation between CRP levels measured before cardioversion and AF, and therefore CRP cannot be considered pathogenic for AF and does not help predict postoperative AF^[21,24]^. A retrospective study published by Uguz B et al.^[21]^, which was conducted in 116 CABG patients, reported that postoperative AF was developed in 22.4% of the patients and preoperative lymphocyte (103/µL) value was found to be 2.5 (1-5.3) and 1.3 (0.4-2.8) in the postoperative AF group and postoperative non-AF group, respectively (*P*<.001); also, the preoperative CRP (mg/L) value was 6.8 (0.6-121.1) and 6.6 (1.2-67.1) in their study, respectively, it and was not statistically significant (*P*=0.866). In the same study, it was reported that CRP value at the 72nd postoperative hour was found to be 61.6 (1.4-327.4) and 49.8 (9.4-219.9), respectively, and it was not found to be statistically significant (*P*=0.369)^[21]^. Similarly, in our study, preoperative CRP values (mg/l) were determined as 8.60 ± 16.84 in the new postoperative AF group and 15.56 ± 23.38 in the group without new AF, and no statistical difference was detected (*P*=0.478).

It has been reported that albumin cells are important because of their anti-inflammatory and anticoagulant features^[4,25]^ and that hypoalbuminemia may be a marker for the development of arterial atherosclerosis and thrombosis, but it has also been reported that hypoalbuminemia associated with malnutrition is an independent predictor of some cardiovascular diseases such as CAD^[1,4,26]^.

PNI, calculated by albumin and lymphocyte values and a valuable malnutrition parameter, was reported as a possible risk factor for postoperative AF^[4]^. There are studies reporting that PNI can also be used as a predictor of prognosis and poor results in patients undergoing CABG^[4,27]^. In a recent study in the literature, the importance of PNI value in patients undergoing CPB-guided heart surgery was expressed, and it was reported that patients were divided into two groups based on PNI value (< 46.13 and > 46.13; PNI value < 46.13 = high risk) and that early-term undesirable events were higher in the lower PNI group^[4,28]^. PNI was also expressed to be a predictor of early-term mortality^[4]^. In light of these results, it was reported that the easily calculable preoperative PNI value is a precious parameter for predicting early-term results in CPB-guided open-heart surgery operations^[4,28]^. In a study, PNI was found to be a predictor of AF development in the postoperative CABG period and it was also reported that old age and chronic obstructive pulmonary disease were also predictors^[4,17]^. In our study, although the mean preoperative lymphocyte and PNI values were found to be lower in the group that developed postoperative AF compared to the other group, this was not statistically significant (*P*=0.084, *P*=0.877, respectively). The only statistically significant parameter in terms of postoperative AF development in both groups was cross-clamping time compared to our study (Group 1: 88.96 ± 16.51 min, Group 0: 81.24 ± 24.5min, *P*=0.008). In addition, although not statistically significant, lymphocytes and albumin, whose anti-inflammatory effects are known and whose predictive roles in terms of postoperative adverse events have been reported in some publications, and the PNI value in their formulation were lower in the group that developed postoperative AF^[4,9]^. We investigated the effect of PNI on postoperative adverse events and AF in our study. There was no statistically significant difference between the patient groups in our study in terms of the patients’ weight (*P*=0.171) and no statistically significant difference between the PNI values (*P*=0.877). However, although not statistically significant, the mean PNI values of patients in the AF group were lower than those in the no-AF group (50.91 ± 5.29 *vs.* 52.07 ± 5.55, respectively). In addition, the group of patients with a PNI value < 51.76 ± 5.48, which was the mean PNI value in our study, had a higher rate of newly developed AF compared to the other group (30 ± 0.46% *vs.* 23 ± 0.42%, respectively).

It has been stated that in the presence of similar risk factors, there are significant differences in the development of new AF in some patients, and the reasons for this are still unknown^[13]^. It has been reported in several studies a potent and independent relation between an increase in some inflammatory markers and new-onset AF in the postoperative CABG period^[13,29]^, on the other hand, it has been expressed that there is a lack of data on the relationship between nutritional status and long-term results and important undesirable events in isolated CABG patients^[1]^. Again, although there are publications stating that they are effective in predicting mortality in CABG patients, it is also stated that there is insufficient data on the relationship between nutritional status and long-term results and important undesirable events in isolated CABG patients^[1]^. In our study, although the mean preoperative lymphocyte and PNI values were lower in the group with postoperative AF compared to the other group, this was not statistically significant (*P*=0.084, *P*=0.877, respectively). In addition, cross-clamping time was longer in patients with new postoperative AF compared to patients without AF (88.96 ± 16.51 min. *vs.* 81.24 ± 24.5 min., respectively, *P*=0.008) in our study.

In a study conducted in patients undergoing off-pump CABG, it was reported that patients in the newly developed AF group were older than patients who did not develop AF, and platelet and systemic immune-inflammation index levels were found to be significantly higher in multivariate analysis among white blood cell, platelet, neutrophil, neutrophil/lymphocyte ratio, and systemic immune-inflammation index levels^[5]^. In our study, no significant statistically difference was found between the mean ages of the patients in both groups, but the male sex ratio was higher in the newly developed AF group compared to the other group (84 ± 0.37% *vs.* 74 ± 0.44%, respectively).

### Limitations

Our article has limitations as a relatively small sample size, a single center, short follow-up period, and a retrospective design. Another limitation of the study is that some possible risk factors for the development of AF were excluded from the evaluation due to the inaccessibility of the information on these risk factors in the hospital automation system.

## CONCLUSION

As a conclusion, although it has been stated in some studies in the literature that there is a relationship between low PNI values and the development of AF after CABG, according to our study results, there was no statistically significant difference between PNI values and the development of AF after CABG, which is similar to some publications in the literature. There are publications in the literature that are compatible or not compatible with our study results and considering that our study was conducted with a relatively low number of patients, we think that it would be beneficial to conduct studies involving more patients focused on this subject.
